# Transcriptional profiling of early differentiation of primary human mesenchymal stem cells into chondrocytes

**DOI:** 10.1038/s41597-023-02686-y

**Published:** 2023-11-03

**Authors:** Thomas Schwarzl, Andrea Keogh, Georgina Shaw, Aleksandar Krstic, Elizabeth Clayton, Desmond G. Higgins, Walter Kolch, Frank Barry

**Affiliations:** 1 European Molecular Biology Laboratory (EMBL), Meyerhofstraße 1, 69117 Heidelberg, Germany; 2 Previously: Regenerative Medicine Institute (REMEDI), Biosciences, National University of Ireland Galway, University Road, Galway, Ireland; 3 Regenerative Medicine Institute (REMEDI), Biosciences, National University of Ireland Galway, University Road, Galway, Ireland; 4 Systems Biology Ireland (SBI), School of Medicine, University College Dublin, Dublin, 4 Ireland; 5 Conway Institute of Biomolecular and Biomedical Research, University College Dublin (UCD), Belfield, Dublin, 4 Ireland

**Keywords:** Mesenchymal stem cells, Stem-cell differentiation

## Abstract

Articular cartilage has only very limited regenerative capacities in humans. Tissue engineering techniques for cartilage damage repair are limited in the production of hyaline cartilage. Mesenchymal stem/stromal cells (MSCs) are multipotent stem cells and can be differentiated into mature cartilage cells, chondrocytes, which could be used for repairing damaged cartilage. Chondrogenesis is a highly complex, relatively inefficient process lasting over 3 weeks *in vitro*. Methods: In order to better understand chondrogenic differentiation, especially the commitment phase, we have performed transcriptional profiling of MSC differentiation into chondrocytes from early timepoints starting 15 minutes after induction to 16 hours and fully differentiated chondrocytes at 21 days in triplicates.

## Background & Summary

Cartilage is a tissue of critical importance in the normal function of the axial skeleton and limb joints, providing the lubricating and shock-absorbing properties of diarthrodial joints and the spine. It also plays a critical role in embryonic limb formation and postnatal growth. Cartilage is a highly specialized avascular and aneural tissue with limited repair capacity^1^. It consists of distinct populations of chondrocytes and an abundant extracellular matrix rich in collagens and proteoglycans^2,3^. Traumatic injury of cartilage and degenerative diseases that lead to tissue loss are common and are major causes of disability and loss of mobility. Therapeutic options are limited and there is currently no regenerative treatment that leads to complete restoration of healthy tissue. Thus, there is a compelling need for new therapeutic paradigms and for effective models to understand the regulation of cartilage differentiation and the pathological mechanisms of disease.

Mesenchymal stem/stromal cells (MSCs) represent one such treatment option that has gained much attention in recent years^4^. In 2018, more than 200 clinical studies using MSCs for cartilage repair were reported^5^. Compelling data from preclinical studies and from early stage human trials indicate that MSC treatment is a potential disease-modifying approach^5,6^. This often involves the delivery of undifferentiated MSC preparations by intra-articular injection. Autologous chondrocyte (ACI) transplantation is also commonly used as a treatment for acute focal cartilage injury, with good outcomes. This approach has some disadvantages, namely the need to surgically harvest a tissue biopsy from the articular surface for chondrocyte isolation and the limited expansion capacity of primary cultured chondrocytes. MSCs are multipotent progenitor cells isolated from a wide range of adult tissues^7^. For therapeutic use the main tissue sources are bone marrow^8^, adipose tissue^8,9^, umbilical cord^10,11^ and placenta^12^. They are readily expandable through many generations in adherent culture using either planar or bioreactor configurations. They possess a well-described trilineage differentiation propensity and can form cartilage, adipose and bone tissues *in vitro* and when transplanted *in vivo*^13^. The use of chondrocytes derived from MSCs is an attractive option for cartilage repair treatments and overcomes the obstacles associated with using primary chondrocytes^14^. MSCs are also tolerated as allogeneic therapy, and several advanced therapeutics consisting of allogeneic MSCs have been approved in recent years^15,16^.

In order to improve the potential therapeutic properties of MSCs for cartilage repair, it is important to understand how and when MSCs commit to chondrogenic differentiation. Of particular interest is the early response to differentiation signals and commitment phase where interventions could be used to increase the number of differentiating cells. Therefore, we examined gene expression during the time-course of chondrogenic differentiation and identified gene regulatory networks (GRNs) that control commitment and differentiation. An obstacle to such studies is the heterogeneity of culture expanded MSC preparations, which consist of mixtures of cells of varying differentiation potential. Therefore, we prepared clonal populations of MSCs from human bone marrow by limiting dilution and obtained a clone with good growth characteristics and clear tri-lineage differentiation potential.

**Fig. 1:**
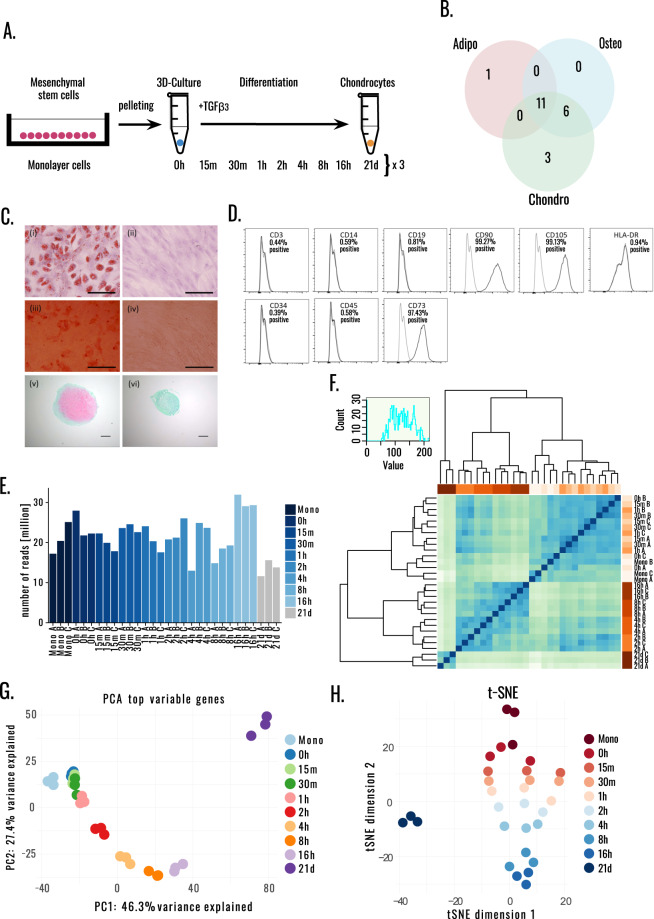
Overview and characterization of a hMSC clone with trilineage differentiation potential. (**A**) Overview of experimental design. Three replicates for Monolayer MSCs, undifferentiated MSCs in 3D culture (0 h) and 15 m, 30 m, 1 h, 2 h, 4 h, 8 h, 16 h after initiation of differentiation, as well as differentiated chondrocytes (21 days after initiation of differentiation). (**B**) Schematic of the trilineage differentiation capacity from the bone marrow derived MSC clones from a single donor. (**C**) The differentiation assays of the clone 1F3 selected for studying the commitment to chondrogenesis. Adipogenic differentiated (i) and control (ii) wells were stained with Oil Red O, with lipid stained vesicles present in the differentiated wells, scale bar 50μm. Osteogenic differentiated (iii) and control (iv) wells were stained with Alizarin Red, and calcium deposition was noted by positive staining in the differentiated wells, scale bar 200μm. Chondrogenic pellets were stained with Safranin O/Fast Green FCF, with positive staining for glycosaminoglycan seen in the differentiated pellets (v) compared to the control pellets (vi), scale bar 100 μM. (**D**) Surface profile analysis of clonal cells was carried out using flow cytometry. The clonal population was negative for CD3, CD14, CD19, CD34, CD45 and HLA-DR, but positive for the MSC markers CD73, CD90 and CD105. Isotype controls are shown in gray, and the black line represents each antigen. (**E**) Number or reads sequenced for each sample, for Monolayer MSCs (Mono), undifferentiated MSCs in 3D culture (0 h) and 15 m, 30 m, 1 h, 2 h, 4 h, 8 h, 16 h after initiation of differentiation, as well as differentiated chondrocytes (21 days after initiation of differentiation) replicated in three batches A, B and C. (**F**) Hierarchical clustering, (**G**) PCA plot of variance stabilized counts from top 1000 genes. (**H**) t-Distributed Stochastic Neighbor Embedding with perplexity 5 and max iter 5000.

A clonal human MSC line was derived from human bone marrow by limiting dilution cloning as described in the Methods section and validated as described in the Technical Validation section. The clone 1F3 cells, which retained full capacity of chondrogenic, adipogenic and osteogenic differentiation, were induced to differentiate by transferring them from monolayer into 3D culture by gently pelleting the cells and adding TGF-β3. RNA was extracted in triplicates from undifferentiated MSCs in monolayer, undifferentiated MSCs in 3D culture (0 h) and 15 m, 30 m, 1 h, 2 h, 4 h, 8 h, 16 h after adding TGF-β3, and terminally differentiated chondrocytes (21 days after differentiation) (Fig. 1A). Transcriptional profiling in chondrogenesis was previously studied with microarrays in human MSCs^17^ and with bulk and single-cell RNA-seq in induced pluripotent stem cells (hiPSCs)^18^. To the best of our knowledge, this is the first bulk RNA-seq transcriptomic study of very early chondrogenesis of bone-marrow derived mesenchymal stem cells.

## Methods

### Isolation and expansion of bone marrow hMSC clones

hMSCs were isolated from a heparinized bone marrow aspirate harvested from the iliac crest of a healthy 23 year old male volunteer. hMSCs were isolated *in vitro* by direct plating as described previously^19^. Cells were plated at a density of 5 × 10^4^ mononuclear cells per cm^2^ in alpha MEM (Gibco) supplemented with 10% fetal bovine serum (FBS; Hyclone) and 1 ng/mL FGF2 (Peprotech). After five days, when colonies were visible, cells were trypsinized and cloned by limiting dilution into 96 well plates (0.3 cells/well), these clones were termed P1. Wells with single clones were passaged and expanded to P4. The trilineage differentiation potential of the clonal populations were examined, which showed clones with differing propensity for differentiation to the chondrocytic, osteoblastic and adipocytic lineages. A single clone, termed 1F3, with trilineage potential was selected for the study of commitment to chondrogenesis. This clone, as well as having the ability to differentiate to osteoblasts, adipocytes and chondrocytes, also displayed the typical fibroblastic-like morphology and surface marker profile of an MSC.

### Characterisation of MSC clones using trilineage differentiation and surface marker expression

MSC clones were characterised by multi-lineage differentiation and flow cytometry for cell surface marker expression. The multi-lineage differentiation capacity of the MSC clones was assessed by their differentiation to the classical MSC lineages i.e., chondrogenic, osteogenic and adipogenic using established protocols^19^.

Chondrogenic potential was examined using a 3-D pellet culture system, where, 2.5 × 10^5^ cells were pelleted at 100 xg for 5 minutes in complete chondrogenic medium (CCM) consisting of Dulbecco’s Modified Eagle Serum (DMEM, 4.5 g/L glucose), 2 mM glutamine, 100 mM dexamethasone, 50 μg/mL ascorbic acid, 40 μg/mL L-Proline, 1% ITS + (Insulin, Transferrin, Selenium) (Corning), 1 mM sodium pyruvate and penicillin-streptomycin (100 U/mL), supplemented with 10 ng/mL TGF-β3 (Peprotech). Control pellets were incubated without TGF-β3 incomplete chondrogenic medium (ICM). After 21 days, pellets were fixed in 10% neutral buffered formalin, and processed for histology in a Leica ASP300S tissue processor and embedded in paraffin. Sections were stained with Safranin O and Fast Green FCF and imaged with an Olympus BX43 microscope.

For osteogenesis, cells were seeded in culture medium and when the monolayer reached 90% confluence, the medium was replaced with osteogenic medium containing DMEM (1 g/L glucose; Sigma Aldrich), 2 mM l-glutamine, 100 nM dexamethasone, 100 μM ascorbic acid, 10 mM β-glycerophosphate, 10% FBS (Hyclone) and penicillin-streptomycin (100 U/ml). Medium was replaced every 3–4 days for up to 14 days, when monolayers were fixed with 10% ice cold methanol, then stained with 2% Alizarin Red and imaged using an Olympus IX71 microscope.

Adipogenesis was assessed by incubating confluent cultures in adipogenic induction medium comprising DMEM (4.5 g/L glucose), 2 mM l-glutamine, 10% FBS (Hyclone), 1 μM dexamethasone, 10 μg/mL insulin (Roche), 200 μM indomethacin, 500 μM 3-isolbutyl-1-methylxanthine and penicillin-streptomycin (100 U/mL). After 3 days, the culture was transferred to adipogenic maintenance medium comprising DMEM (4.5 g/L glucose), 10% FBS (Hyclone), 10 μg/mL insulin (Roche) and penicillin-streptomycin (100 U/mL) for 1 day. This cycle was repeated 3 times after which the cells were maintained in adipogenic maintenance medium for a further 5 days. Cultures were fixed in 10% neutral buffer formalin and stained with Oil Red O before imaging using an inverted Olympus IX71 microscope.

Surface marker expression of MSC clones was carried out by flow cytometry using the BD FACS Canto II flow cytometer (BD Biosciences) using antibodies against CD3, CD14, CD19, CD34, CD45, HLA-DR and the MSC positive markers CD73, CD90 and CD105 (BD Biosciences) as described previously^20^. Post-acquisition analysis was carried out using the FlowJo software (Treestar Inc.).

### Study of the commitment towards chondrogenesis

To examine the commitment towards chondrogenesis, clonal cell cultures were switched to a culture medium containing 1% FBS twelve hours prior to chondrogenic induction. Chondrogenic differentiation was carried out in a pellet culture format as described above. Three pellets were set up for each time point. Pellets from the 0 h time point remained in ICM, while pellets at 15 min, 30 min, 1, 2, 4, 8 and 16 h time points were cultured in CCM. At each time point the 3 pellets were combined into 1 tube, the pooled pellets were washed in PBS and snapped frozen in liquid nitrogen. Clonal cells in monolayer and 21d chondrogenic pellets were included as controls. The time course experiment was carried out three times and each time course served as a technical replica for the experiment.

### RNA extraction and sequencing

Total RNA was extracted using an miRNeasy Mini kit (QIAGEN). For day 21 samples, due to the amount of matrix deposition around the chondrocytes in this late time point pellet, mechanical homogenisation by pulverizing in a chilled steel mortar and pestle was required prior to RNA extraction^21^. The concentration and purity of the isolated RNA was determined using the Nanodrop ND-1000 (Nanodrop Technologies). RNA integrity was measured using the 2100 Bioanalyzer (Agilent Technologies). Bioanalyzer and Nanodrop results were deposited on Zenodo as lab_qc.zip^22^. 400 ng of total RNA was included in each sequencing reaction.

Poly(A)+ enrichment to purify mRNA from each sample was performed by hybridization of the RNA with poly(T) oligomers. The mRNA was then converted to cDNA and fragmented, creating cDNA library fragments of approximately 250nt in size. Sequencing adaptors were then added to each cDNA library fragment. The libraries were sequenced on an Illumina GAIIx using TruSeq Single read Cluster Generation Kit v5 and TruSeq Single read 36 cycle Sequencing kit v5. The sequencing mode was 35 nt single-read plus 7 nt for indices. The raw reads were aligned to Human Gencode^23^ (v23/h38.p3) reference genome with STAR aligner^24^ (v2.5.0a) and summarized with featureCounts^25^ (v.1.6.2).

### Ethics approval and consent to participate

All procedures were approved by the Clinical Research Ethical Committee of Galway University Hospital and by the National University of Ireland Galway Research Ethics Committee, including written consent to participate and publish sequenced data.

## Data Records

### Availability of data and materials

Transcriptional profiling of undifferentiated human MSCs (Mono), MSCs in 3D culture (0 h), early differentiation time-points 15 m, 30 m, 1 h, 2 h, 4 h, 8 h, 16 h after induction with TGF-β3 and hyaluronic acid, and fully differentiated chondrocytes (21 days after induction) using RNA-seq in triplicates of clones was performed. Raw sequencing data (fastq), sample information (MAGE-TAB format) and summarized counts (tab-delimited text file) have been submitted to BioStudies under accession number E-MTAB-10476^26^. MultiQC of FastQC and alignment statistics, PCA and differential gene expression analysis with DESeq2^27^ and edgeR^28^, time-course clustering Mfuzz^29^, BigWig files, function annotation and functional analysis with Ingenuity Pathway Analysis and Cluster Profiler^30^ was deposited on Zenodo^22^.

## Technical Validation

### Selection of 1F3 clone

In total 21 clones were isolated from one donor, of which 11 had trilineage capacity (Fig. 1B). The selected clone 1F3 retained the full capacity of MSCs for chondrogenic, adipogenic and osteogenic differentiation (Fig. 1C). Surface marker expression of the MSC clone was carried out by flow cytometry using PE labelled antibodies (BD Biosciences) against CD3, CD14, CD19, CD34, CD45, HLA-DR and the MSC positive markers CD73, CD90 and CD105 as described previously^19^. Antibody details are supplied in supplementary^22^. Cells were washed and blocked in PBS with 2% FBS prior to incubation for 30 minutes with antibodies, diluted to the recommended concentrations according to the manufacturer’s instructions. Following washing, Sytox Red (Invitrogen), a stain for dead cells was added prior to acquisition of the samples on a BD FACS Canto II (BD Biosciences), where the live single cell population was analyzed. Post-acquisition analysis was carried out using the FlowJo software (Treestar Inc.). Flow cytometric analysis of cell surface antigens showed that the clones were positive for MSC markers CD105, CD90 and CD73 and negative for hematopoietic markers CD45, CD34, CD19, CD3 and CD14, with minor expression of HLA-DR (Fig. 1D).

### Bioinformatics Validation of Sequencing data

The raw reads were aligned to Human Gencode^23^ (v23/h38.p3) reference genome with STAR aligner^24^ (v2.5.0a) and summarized with featureCounts^25^ (v.1.6.2) and are available in BioStudies^26^. The median library size is 21.52 million reads (Fig. 1E). The three differentiated (21-days) samples have 13.69 million reads on average, due to technical challenges of RNA extraction from differentiated chondrocytes. Hierarchical clustering (Fig. 1F), Principal component analysis (PCA) (Fig. 1G) of the top thousand variable genes as well t-SNE confirming that the experimental conditions are very reproducible (Fig. 1H).

## Usage Notes

Functional annotation, differential gene expression analysis, time-course clustering and upstream regulator prediction workflow can be found at Zenodo^22^.

## Data Availability

A compiled protocol and supplementary files and information are deposited as ‘report.zip’ at Zenodo^22^. The index.html links to the analysis report and compiled R markdown file. The raw code and source annotations are deposited as ‘analysis.zip’ in the same repository.
